# Analysis of potential biomarkers of response to IL‐12 therapy

**DOI:** 10.1002/JLB.5RU1221-675R

**Published:** 2022-07-04

**Authors:** Emily Schwarz, William E. Carson

**Affiliations:** ^1^ Biomedical Sciences Graduate Program, College of Medicine The Ohio State University Columbus Ohio USA; ^2^ Comprehensive Cancer Center The Ohio State University Columbus Ohio USA; ^3^ Department of Surgery, Division of Surgical Oncology The Ohio State University Columbus Ohio USA

**Keywords:** cells, cytotoxic T cells, myeloid suppressor cells, NK oncology, chemokines, cytokines, immunotherapy

## Abstract

IL‐12 is a proinflammatory cytokine capable of inducing a wide range of effects on both innate and adaptive immune responses. Its stimulatory effects on T cells and NK cells have led to its classification as a potential inducer of antitumor immunity. Clinical trials have been attempting to harness its immune‐stimulating capacity since the 1990s and have had much success despite notable toxicity issues early on. Several methods of IL‐12 delivery have been employed including i.v., s.c., and local administrations as well as plasmid and gene therapies. However, despite differing methods, dosages, and cancer types utilized in these clinical trials, there are still many patients who do not respond to IL‐12 therapy. This creates an opportunity for further investigation into the immunologic differences between responding and nonresponding patients in order to better understand the variable efficacy of IL‐12 therapy. This review focuses on a limited collection of IL‐12 clinical trials, which further analyzed these individual subsets and detected biologic variables correlating with differential patient responses. A comprehensive review of these potential biomarkers identified 7 analytes that correlated with beneficial patient responses in 3 or more clinical trials. These were increased levels of IFN‐γ, IP‐10, TNF‐α, MIP‐1α, MIG, and CD4^+^ and CD8^+^ T cells, with a decrease in VEGF, bFGF, FoxP3^+^ T regulatory cells, and M2 macrophages. These potential biomarkers highlight the possibility of identifying immunologic determinants of patient response to IL‐12 therapy to conserve valuable resources and benefit patients.

## Graphical Abstract

Review of potential correlates of beneficial patient response to IL‐12 therapy in the treatment of cancer.

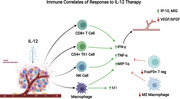

**Abbreviations**
ADCC: antibody‐dependent cellular cytotoxicity
Ad‐RTS‐mIL‐12: replication‐incompetent adenoviral vector engineered to express IL‐12 via a RheoSwitch Therapeutic System
bFGF: basic fibroblast growth factor
CD: cluster of differentiation
CR: complete responder
CTCL: cutaneous T cell lymphoma
EGFR: epidermal growth factor receptor
GEN‐1/EGEN‐001: IL‐12 plasmid formulated with PEG‐PEI‐cholesterol lipopolymer
HD: Hodgkin's disease
HER: human epidermal growth factor receptor
HLA: human leukocyte antigen
i.l: intralesional
i.t.: intratumoral
IHC: immunohistochemistry
IP: IFN‐gamma‐inducible protein
MAGE‐A: melanoma‐associated antigen
MART: melanoma‐associated antigen recognized by T cells
MCC: Merkel cell carcinoma
MCPyV: Merkel cell polyomavirus
MDSC: myeloid‐derived suppressor cell
Mib: Mindbomb
MIG: monokine induced by gamma
MIP: macrophage inflammatory protein
MTD: maximally tolerated dose
NHL: non‐Hodgkin's lymphoma
PD‐L: programmed death‐ligand
PEC: peritoneal exudate cells
PFS: progression‐free survival
pIL12: tavokinogene teleplasmid
PR: partial responder
R: receptor
rh: recombinant human
rm: recombinant mouse
s.c.: subcutaneous
sFasL: soluble Fas ligand
T‐bet: T‐box expressed in T cells
TIA‐1: T cell‐restricted intracellular antigen
Treg: T regulatory cell
TYK: tyrosine kinase
VDX: veledimex
VEGF: vascular endothelial growth factor

## 1 INTRODUCTION

IL‐12 is a heterodimeric cytokine that is capable of inducing a wide range of immune effects. It is a member of the larger IL‐12 family, which also includes 3 other cytokines; IL‐23, IL‐27, and IL‐35. IL‐12 itself is composed of one 35‐kDa alpha‐chain subunit (p19 or p35) paired with one 40‐kDa beta‐chain subunit (p40).^1^ It is known as a predominantly proinflamamtory cytokine and can be produced by several different cell types including monocytes, macrophages, dendritic cells, neutrophils, and B cells.^2^ IL‐12 signaling occurs through the IL‐12 receptor (IL‐12R), which consists of 2 type I transmembrane glycoprotein chains; ∼100 kDa IL‐12Rβ1 and ∼130 kDa IL‐12Rβ2. IL‐12Rβ2 acts as the signal‐transduction component by inducing phosphorylation of JAK‐STAT proteins downstream.^3^ More specifically, activation of IL‐12R causes tyrosine phosphorylation of primarily JAK2 and TYK2, which can then phosphorylate STAT1, STAT3, STAT4, and STAT5.^2^, ^4^ Although IL‐12R is mainly expressed on activated NK and T cells, it has also been found on dendritic cells and B‐cell lines.^5^, ^6^ The widespread expression of the IL‐12R allows for the pleiotropic effects of IL‐12 on both innate and adaptive immune cells. For example, IL‐12 stimulation of NK and T cells induces production of IFN‐γ through STAT4 activation.^7^ IFN‐γ can then further activate macrophages, NK cells, and B cells and up‐regulate T‐bet to promote a Th1 T cell response.^8^ Furthermore, the ability of IL‐12 to stimulate T cells and NK cells has made it an attractive candidate for overcoming the immunosuppressive microenvironments identified in several disparate types of cancer.^9^, ^10^, ^11^

The potential for IL‐12‐induced antitumor immunity led to its identification as a promising therapeutic agent for the treatment of cancer. After several successful preclinical models demonstrated the benefit of IL‐12 for the treatment of cancer, this cytokine therapy progressed into human clinical trials in the 1990s. One of the first clinical trials utilizing recombinant human (rh) IL‐12 was reported by Atkins et al.^12^ in 1994. This phase I trial enrolled 40 patients with renal cancer, melanoma, or colon cancer. The primary objective was to monitor the toxicity and effectiveness of bolus i.v. rhIL‐12. Cohorts of 4–6 patients were treated with doses escalating from 3 to 1000 ng/kg. The regimen included an inpatient test dose, a 2‐week rest period, and then consecutive daily outpatient doses for 5 days every 3 weeks. This allowed for the establishment of 500 ng/kg as the maximally tolerated dose (MTD) and resulted in 1 partial response, 1 transient complete response, and 4 patients with no disease progression. IFN‐γ was induced in a dose‐dependent fashion with mean serum levels exceeding 1000 pg/ml at 24 h in patients receiving the 1000 ng/kg test dose. Levels returned to a baseline of 0 pg/ml by day 8 postdosing. Mean IFN‐γ levels then increased to their peak concentrations of approximately 16,500 pg/ml for the 1000 ng/kg cohort during days 3–4 of the first consecutive 5‐day treatment period, with peak concentrations progressively declining after each successive treatment cycle. Serum neopterin levels reached a peak of approximately 225 nmol/L at day 5 and then subsequently declined. TNF‐α serum levels were undetectable at the time points measured; however, the occurrence of fever and chills in patients prior to sample collection might have been indicative of a transient up‐regulation. Further investigation into the immunologic effects of this treatment regimen in 4 patients treated at the 500 ng/kg dose level revealed peripheral blood NK cells expressed significantly higher surface densities of CD2, CD11a, and CD56 3 to 4 days after rhIL‐12 infusion. Furthermore, NK cell cytotoxicity toward K562 leukemia target cells was significantly increased in PBMCs isolated 3–7 days after dosing compared with cells isolated pretreatment. A significant increase in PBMC proliferation induced by immobilized anti‐CD3 monoclonal antibody was also detected in vitro in PBMCs isolated after a single injection of 500 ng/kg rhIL‐12.^13^

These encouraging safety and biologic responses led to a follow‐up phase II trial by the same group that administered rhIL‐12 at the MTD of 500 ng/kg by bolus i.v. once daily for 5 consecutive days every 3 weeks.^14^ However, severe toxicities occurred with 2 deaths and 12 of the 17 treated patients requiring hospitalization. An investigation was subsequently initiated to determine the cause of these unexpected results and a pause was placed on the use of IL‐12 in clinical trials.^15^ This action created some hestancy toward the safety of IL‐12 therapy. The investigation determined that the test dose had in fact abrogated toxicity and that its omission in the phase II study had contributed to the observed toxicity of the regimen.^14^

Follow‐up studies were then conducted by Leonard et al.^14^ in 1997 to investigate a potential link between IL‐12‐induced IFN‐γ production and IL‐12‐induced toxicity in the phase II trial. After determining that there were no substantial changes in the drug products used during the 2 trials, focus shifted toward the change in schedule of IL‐12 administration. Complementary experiments were performed in C3H/Hej mice. First, mice were treated with 0.5 or 1.0 µg rmIL‐12 for 6 consecutive days without pretreatment. This schema resulted in 100% mortality by day 8 and adverse events such as gastrointestinal toxicity and diarrhea, consistent with those events seen in the phase II trial patients. Pretreatment with a single dose of 0.5 µg rmIL‐12 a week prior to consecutive dosing however completely abrogated treatment‐associated toxicity and prevented death. Consecutive daily rmIL‐12 administration to mice without pretreatment resulted in a marked increase in serum IFN‐γ levels that peaked 3–4 days later. Conversely, pretreated mice displayed substantial attenuation of serum IFN‐γ levels between days 2 and 4. In order to determine if this phenomenon mirrored what had occurred in phase II clinical trial patients, serum samples collected from patients receiving 500 ng/kg i.v. rhIL‐12 in both trials were assayed for IFN‐γ levels. Samples from phase II clinical trial patients showed substantially higher mean IFN‐γ levels with a peak of approximately 27,000 pg/ml on day 4 compared with those from the phase I trial that had a considerably lower peak of approximately 5000 pg/ml on day 3. TNF‐α was not detected in the serum of patients from either trial, ruling out the contribution of this additional IL‐12‐induced cytokine. Thus, in order to confirm the potential link between increased IFN‐γ levels and toxicity issues, mice were treated for six consecutive days with 0.5 µg rmIL‐12 in the presence of either an IFN‐γ neutralizing antibody or a control antibody. The mice treated with control antibody developed severe toxicity and exhibited 100% mortality after 7 days while IFN‐γ neutralization protected IL‐12 treated mice from toxicity with 0% mortality. These results were confirmed by corresponding studies in cynomolgus monkeys and allowed for the determination that IL‐12‐induced toxicity issues may be overcome by controlling the IFN‐γ response. Furthermore, it was determined that the single test‐dose prior to consecutive IL‐12 treatment was capable of controlling the plasma IFN‐γ response and abrogating the severe toxicities that previously hindered clinical use of IL‐12. Based on this improved understanding of IL‐12 biology and the effect of different treatment schedules, there has been a renewed interest in its therapeutic use.^16^ Current approaches to IL‐12 therapy capitalize on recent protein engineering advancements and utilize IL‐12‐based molecules with enhanced localization and retention.^17^ These molecules include IL‐12 fusion proteins or “immunocytokines,” which allow for increased targeting of tumor antigens or extracellular matrix proteins, cell surface‐tethered IL‐12 molecules, IL‐12 molecules fused to binding domains and IL‐12 molecules with increased circulatory stability.^18^, ^19^, ^20^, ^21^ Additionally, IL‐12 molecules capable of targeting specific T cell populations are also being developed to improve antitumor responses. For example, Li et al.^22^ fabricated a dual‐target immune‐nanoparticle encasing IL‐12 and expressing CD8 and glypican‐3 antibodies on the surface, allowing for targeted CD8+ T cell delivery. This targeted IL‐12 delivery method was found to increase the expansion, activation, and cytotoxicity of CD8+ T cells crucial for antitumor responses.

The focus on IL‐12 in this review is a reflection of the renewed interest in this cytokine given the advanced technology that is being used to administer it in a more localized and physiologic manner. Moreover, the present review was conducted due to the increased clinical usage of IL‐12 in the setting of cancer therapy.^17^, ^23^ A subset of IL‐12 clinical trials have also used correlative studies to investigate the biologic effects of IL‐12‐induced immunity in patients with favorable clinical responses. This information has allowed for the identification of several potential correlates of response to IL‐12 therapy. These correlates may serve as useful biomarkers allowing for more precise monitoring of patient responses during future IL‐12 studies. In addition, they could provide a better understanding of IL‐12‐induced immunity. This review will go on to summarize the correlates of response that have been identified to date.

## 2 CORRELATIVE STUDIES CONDUCTED ON PATIENTS RECEIVING i.v. IL‐12

Several methods of IL‐12 administration have been employed in an effort to determine the optimal delivery system for patient benefit. While advancements in technologies have developed over time leading to more localized and controlled administration, the use of systemic delivery by the i.v. route has been utilized by several groups. For example, i.v. rhIL‐12 as a single agent therapy was evaluated for the treatment of patients with metastatic renal cell cancer or malignant melanoma in a phase I clinical trial conducted in 2000. Gollob et al.^24^ explored the effects of i.v. bolus rhIL‐12 administered twice weekly for 6 weeks at increasing doses of 30, 100, 300, 500, or 700 ng/kg. There were no responses in any of the 19 patients treated below the MTD of 500 ng/kg. Of the remaining 9 patients, one experienced a partial response at 500 ng/kg and 3 experienced disease stabilization; 2 patients at 500 ng/kg and 1 patient at the 700 ng/kg dosage level. Plasma levels of IL‐12 and IL‐10 were measured and PBMCs were assayed for in vitro production of IFN‐γ and levels of thymidine incorporation in response to stimulation with IL‐12 alone or in combination with IL‐2 or IL‐15. Further analysis was conducted on specimens from eight of the patients treated at 500 ng/kg and 2 patients treated at 700 ng/kg to quantify IFN‐γ, IL15, and IL‐18 plasma levels postinjection. Three discernable patterns of IFN‐γ induction were found among these 10 patients. These included a type‐1 pattern, defined as peak IFN‐γ levels rising to approximately 450–1600 pg/ml after the first dose, rising an additional 2–3‐fold higher after the second dose, and returning to around 450–1600 pg/ml following the seventh dose; a type‐II pattern, defined as peak levels after the first dose averaging 2‐fold higher than type‐I patients, remaining or increasing another 2‐fold after the second dose, and decreasing following the seventh dose; and a type‐III pattern, defined as a modest peak in levels following the first dose with continuingly decreasing levels following the second and seventh doses. An association was also found between the ability to sustain IFN‐γ levels and the ability to sustain circulating IL‐15 levels. Patients unable to sustain IFN‐γ levels, classified as type‐II and type‐III induction patterns, all had progressive disease. Conversely, all of the patients who experienced either a partial response or disease stabilization were classified as having a type‐I pattern of IFN‐γ induction. Moreover, as opposed to PBMCs from nonresponders, which lost proliferative response and IFN‐γ induction from IL‐12, IL‐2, and IL‐15 by week 4, PBMCs from responding patients demonstrated sustained proliferation and IFN‐γ production during the 4 weeks they were monitored. Considering rhIL‐12 pharmacokinetics were the same in all patients regardless of IFN‐γ induction‐pattern, sustained induction of IFN‐γ over time may be a biomarker of beneficial response to IL‐12 treatment.

Intravenous IL‐12 has also been given in combination with other therapeutic cytokines including IL‐2. In a 2003 trial, Gollob et al.^25^ investigated the efficacy of i.v. rhIL‐12 in combination with low‐dose IL‐2 for the treatment of patients with metastatic melanoma, transitional‐cell cancer of the bladder, or renal cell cancer. rhIL‐12 was given twice weekly i.v. at a dose of either 300 ng/kg or 500 ng/kg in 6‐week cycles with IL‐2 treatment beginning midway through the first cycle. 300 ng/kg rhIL‐12 was combined with s.c. administered IL‐2 at 0.5 MU/m^2^ for the first dose level and in subsequent dosage levels, rhIL‐12 was increased to 500 ng/kg and IL‐2 was increased successively from 0.5 to 1.0, 3.0, or 6.0 MU/m^2^. Twenty‐four patients in total were divided between these 5 dosage levels resulting in 1 patient with a partial response, 8 patients with disease stabilization, and 15 patients with progressive disease. Several analytes were monitored during the course of treatment including plasma levels of IFN‐γ, TNF‐α, IP‐10, IL‐2, IL‐10, macrophage inflammatory protein (MIP‐1α), and soluble (s) Fas ligand (L). Lymphocyte subset levels and cytokine receptor expression were also evaluated by flow cytometry. Overall, disease stabilization for more than one cycle occurred primarily in patients receiving doses of IL‐2 capable of augmenting IFN‐γ levels when combined with rhIL‐12. Moreover, increased infiltration of CD4^+^ and T cell intracellular antigen (TIA)‐1^+^ CD8^+^ T cells was detected in the tumors of one patient who had significant regression after the addition of IL‐2 treatment. This is noteworthy as TIA‐1 is a protein associated with granules in cytotoxic T cells and a decrease in the percentage of TIA‐1+ tumor infiltrating leukocytes has been shown to correlate with tumor progression in malignant melanoma.^26^ Therefore, this study confirms the potential use of increased IFN‐γ levels posttreatment as a correlate of response to IL‐12 gene therapy and the potential importance of increased CD4^+^ and TIA‐1^+^ CD8^+^ T cell tumor infiltration.

A phase I trial conducted in 2004 analyzed the effect of i.v. administered rhIL‐12 in combination with the HER2 protein‐binding monoclonal antibody trastuzumab in patients with metastatic HER2‐positive malignancies (breast, *n* = 12; pancreatic cancer‐2; cervical cancer‐1).^27^ In this study, Parihar et al.^27^ administered a loading dose of trastuzumab at 4 mg/kg, then reduced the dosage to 2 mg/kg given i.v. on day 1 of each weekly cycle. After 3 cycles, i.v. rhIL‐12 was added to the treatment on days 2 and 5 of each cycle in dose‐escalating cohorts of 30, 100, 300, or 500 ng/kg. Out of 15 patients, there was 1 complete response, 2 patients with stable disease, and 12 patients with progressive disease. Several methods were undertaken to investigate these responses including RT‐PCR and intracellular flow cytometry of PBMCs for IFN‐γ, ELISAs for IFN‐γ, TNF‐α, GM‐CSF, MIP‐1α, IP‐10, and MIG plasma levels and a chromium release assay to measure antibody‐dependent cellular cytotoxicity (ADCC) against trastuzumab‐coated, HER2‐overexpressing tumors using patient PBMCs with or without rhIL‐12 restimulation. Donor PBMCs were also analyzed via a cytokine genotyping assay to measure functional polymorphisms within the genes for IFN‐γ, TGF‐β, TNF‐α, IL‐6, and IL‐10. After analysis, measurable levels of IFN‐γ were detectable on days 1, 2, and 5 of the treatment cycle only in the serum of clinically benefitting patients. Moreover, IFN‐γ induction was associated with a significant increase in progression‐free survival (PFS). RT‐PCR of patient PBMCs corroborated this finding as only the 3 clinically benefiting patients exhibited dramatic increases in IFN‐γ transcript induction during days 1 and 2 of the treatment cycle. Detectable levels of IFN‐γ production by NK cells were also only seen in cells isolated from patients who clinically benefitted. Of these 3 clinically benefiting patients, the patient with a complete response was found to be the only one with a T/T IFN‐γ gene polymorphism at residue 847 of intron 1, which is normally associated with high IFN‐γ production. No other cytokine gene polymorphism exhibited any correlation with response. Increases in circulating levels of MIG, IP‐10, TNF‐α, and MIP‐1α were additionally detected only in patients with clinical benefit. Therefore, increased IFN‐γ transcript induction and production by NK cells, in addition to increased MIG, IP‐10, TNF‐α, and MIP‐1α levels, are candidate correlates of response to IL‐12 therapy. However, it should be noted that the addition of rhIL‐12 to trastuzumab did not appear to enhance the antitumor activity of anti‐HER2 antibody therapy in this phase I study.

In 2009, Bekaii‐Sab et al.^28^ explored the combination of i.v. IL‐12, trastuzumab, and paclitaxel in an attempt to improve treatment efficacy in HER2 oncogene overexpressing cancers. This phase I clinical trial included patients with metastatic HER2‐overexpressing cancers (breast, *n* = 7; colon‐6; esophagus‐4; stomach‐2; pancreas‐1; thyroid‐1). Trastuzumab was administered i.v. at 4 mg/kg for the first dose and then at 2 mg/kg for subsequent doses on day 1 of each weekly cycle. Beginning in cycle 2, IL‐12 was given on days 2 and 5 to dose escalated cohorts at 100 ng/kg i.v., 300 ng/kg i.v. or 200 ng/kg s.c. for cohorts 1–3, respectively. Last, paclitaxel was given i.v. at 175 mg/m2 every 3 weeks. Patients responded well to this combination treatment as there was 1 complete response, 4 partial responses, and 6 patients with stable disease lasting more than 3 months out of a total of 21 patients. In order to identify possible correlates of response, patient plasma was analyzed for cytokine, chemokine and antiangiogenic factor levels and PBMCs were analyzed for levels of intracellular IFN‐γ and ERK phosphorylation. IFN‐γ levels were found to correlate with response as its induction was associated with an increase in PFS. Additionally, patients exhibiting clinical benefit had significantly higher peak levels of IP‐10, MIG, MIP‐1α, and IL‐8 compared with patients with progressive disease. A significant relationship was found between the induction of p‐ERK in NK cells and clinical response, as enhanced ERK activation on day 5 occurred only in patients with a clinical response or significant disease stabilization. In this study, clinical responses were only detected after the addition of IL‐12 to the treatment regimen. However, responses were observed in response to both the i.v. and s.c. administration of IL‐12. Of note, a patient with metastatic HER2 3+ esophageal cancer achieved a PR after 8 cycles that lasted for 25 weeks and a patient with HER2 2+ metastatic gastric cancer had stabilization of disease that was maintained for 62 weeks. This result was one of the first instances where HER2‐directed therapy was found to benefit patients with gastrointestinal cancers. These correlative studies confirmed the induction of IFN‐γ as a marker of IL‐12 response and demonstrated the potential role of NK cells in the response to IL‐12 combined with an antitumor monoclonal antibody.

Younes et al. explored the efficacy of i.v. and s.c. delivery of rhIL‐12 in a 2004 study of 42 patients with relapsed and refractory non‐Hodgkin's lymphoma (NHL) or Hodgkin's disease (HD).^29^ Intravenous rhIL‐12 was given to 11 patients daily at 250 ng/kg for 5 days every 3 weeks and s.c. rhIL‐12 was given twice weekly at 500 ng/kg to 31 patients. After completion of the study, 29 NHL patients were evaluable for response with 2 patients having experienced a complete response, 4 patients having partial responses, 10 patients having stable disease, and 13 patients having progressive disease. Additionally, 10 HD patients were evaluable for response with 5 having stable disease and 5 having progressive disease. It is notable that 40% of patients who received rhIL‐12 i.v. had a partial or complete response while just 7% of patients who received rhIL‐12 s.c. exhibited a clinical response. Both NHL and HD patients were then further evaluated for posttreatment changes in peripheral blood lymphocyte counts by flow cytometry and serum levels of vascular endothelial growth factor (VEGF), bFGF, and IFN‐γ by ELISA. Median cell counts of CD4^+^ T cells were slightly increased from 339 to 342/µl following IL‐12 treatment while median cell counts of CD8^+^ T cells were significantly increased from 423/µl to 576/µl after treatment. The median time to peak CD8^+^ T cell counts in these patients was found to be 36 days. Moreover, levels of VEGF and bFGF were found to have increased in multiple patients with progressive disease after treatment with rhIL‐12. Baseline concentrations of VEGF and bFGF were also higher in those patients with aggressive NHL compared with those with indolent disease. Thus, lower pretreatment levels as well as reduced posttreatment levels of VEGF and bFGF may be potential markers of positive response to IL‐12 therapy.

## 3 CORRELATIVE STUDIES CONDUCTED ON PATIENTS RECEIVING s.c. IL‐12

The s.c. route has also been utilized in several IL‐12 studies. Subcutaneous delivery of IL‐12 was employed by Alatrash et al.^30^ in a 2004 trial exploring rhIL‐12 and rhIFN‐α−2b as a combination immunotherapy for patients with renal cell carcinoma or malignant melanoma. In this study, 26 patients were assigned to one of four dosage cohorts and treated in 4‐week cycles. rhIL‐12 was administered s.c. at either 100 ng/kg (dose I group), 300 ng/kg (dose II group), or 500 ng/kg (group III, IV) twice weekly and rhIFN‐α‐2b was given s.c. at 1.0 MU/m2 (dose I, II, and III groups) or 3.0 MU/m2 (dose IV group) thrice weekly. Out of the 4 dosage levels, the dose IV group had 2 partial responders (PRs) (1 malignant melanoma patient, 1 renal cell cancer patient) and dose group I had one such response (renal cell cancer patient). Over the course of treatment, PBMCs were collected for gene expression measurements of IP‐10, MIG, and CD80 by RT‐PCR and T cells were purified for measurement of mRNA expression levels of IFN‐γ and IL‐5. In one of the patients who had a partial response, there was an early and significant increase in both IP‐10 and MIG expression as well as maximum up‐regulation of the costimulatory molecule CD80 on PBMCs after 7 days. While PBMCs from three other patients also showed up‐regulation of CD80, expression peaked after only 6 h in each case. Additionally, the 2 largest fold increases in both IFN‐γ and IL‐5 were seen only in one of the other partially responding patients. Thus, while early increased IP‐10 and MIG expression may be a marker of response to IL‐12 gene therapy, late and continued expression of CD80 may was variably correlated with antitumor activity. Increases in IFN‐γ and IL‐5 may also indicate a possible response to treatment with IL‐12.

In 2019, this same method of s.c. delivery of a combination therapy was explored by McMichael et al.^31^ In their phase I/II trial, this group showed clinical responses in the treatment of patients with epidermal growth factor receptor (EGFR or HER1)‐expressing head and neck squamous cell carcinoma using a combination of s.c. administered IL‐12 and the anti‐EGFR monoclonal antibody cetuximab. In the phase I portion of this study, patients were treated with i.v. cetuximab at 500 mg/m^2^ every 2 weeks in combination with s.c. IL‐12 on days 2 and 5 of a 2‐week cycle at 200 or 300 ng/kg. In the phase II portion of this study, 17 patients were treated with 300 ng/kg IL‐12 with cetuximab. In this phase II portion, 13 patients experienced stable disease lasting for an average of 206 days and 4 had progressive disease. Analysis of serum cytokine and chemokine levels, peripheral blood immune‐profiling, ADCC activity by NK cells and FcγRIIIa polymorphisms led to identification of multiple response correlates. First, there was an increase in ADCC in patients with PFS > 100 days. These same patients also had significantly increased serum levels of IFN‐γ, IP‐10, and TNF‐α at the beginning of cycle 8 compared with baseline. Finally, patients with PFS > 100 days were also found to have a predominance of monocytic myeloid‐derived suppressor cells (MDSC) versus granulocytic MDSC prior to therapy. Therefore, pretherapy profiling of immuno‐suppressive MDSC populations and posttherapy NK cellular activation and circulating IFN‐γ, IP‐10, and TNF‐α levels may correlate with a beneficial response to IL‐12 therapy.

## 4 CORRELATIVE STUDIES CONDUCTED ON PATIENTS RECEIVING IL‐12 VIA LOCAL ADMINISTRATION

In addition to systemic delivery, localized delivery has also been explored as a route of IL‐12 administration. Several methods have been utilized in order to accomplish local dosing including i.p., intralesional (i.l.), and intratumoral (i.t.) injections of IL‐12. One example of a trial that combined s.c. and i.l. administration was the 1999 phase I trial by Rook et al.,^32^ which evaluated rhIL‐12 in 10 patients with cutaneous T cell lymphoma (CTCL) including 5 with extensive plaque, 3 with Sezary syndrome, and 2 with extensive tumors with large cell transformation. In this study, rhIL‐12 was delivered either s.c. or i.l. at escalating doses of 50, 100, or 300 ng/kg twice weekly. There were 2 complete responses, 3 partial responses, 2 local lesion responses, 1 minor response (defined as 25% to 49% disappearance of all CTCL skin lesions for at least 1 month), 1 patient with no response, and 1 patient who withdrew after only 1 week of treatment. T cell responses were analyzed through immunohistochemistry (IHC) staining for CD3, CD4, CD7, CD8, CD25, TIA‐1, HLA‐DR, and Ki67 (proliferative marker) on tumor biopsy samples. In each of the 5 clinical responders (CR, PR), a 2–3‐fold reduction in total T cells mirroring a 2–3‐fold increase in CD8^+^ cytotoxic T cells was observed. In 2 of 3 evaluable cases, a 2–3‐fold increase in TIA‐1^+^ T cells was also detected. These 2 cases also had reduced epidermal HLA‐DR expression and trended toward reduced epidermal hyperplasia, scale, and ulceration. One of the patients with a complete response also had a previously detected TCR gene arrangement that became entirely undetectable following treatment. Based on these results, it was hypothesized that a reduced proportion of total T cells mirrored by increased CD8^+^, TIA‐1^+^ T cells in the tumor and/or lesion might correlate with a positive response to IL‐12 therapy. Changes in TCR gene arrangement subsequent to IL‐12 treatment might be more easily tracked in future studies due to the availability of new RNA sequencing technology.

A study that utilized localized delivery of IL‐12 through i.p. injections was the 2002 phase I clinical trial by Lenzi et al.^33^ This study sought to determine the efficacy of rhIL‐12 as single agent therapy for patients with peritoneal carcinomatosis from Müllerian carcinomas, gastrointestinal tract carcinomas, and peritoneal mesothelioma. To do so, weekly rhIL‐12 was given i.p. at doses of 3, 10, 30, 100, 300, or 600 ng/kg. Out of 29 total patients, 2 patients had complete responses, 8 patients had stable disease, and 19 patients had progressive disease. Levels of multiple cytokines were measured in serum and peritoneal fluid including IL‐1α, IL‐2, IL‐10, TNF‐α, and IFN‐γ. mRNA levels of IFN‐γ, IL‐10, and IP‐10 in peritoneal exudate cells (PEC) were measured by RT‐PCR. bFGF and VEGF expression were measured by IHC and apoptosis was quantified by a terminal deoxynucleotidyl transferase‐mediated nick end labeling assay. Immunofluorescence was used to analyze differentiation markers on peritoneal mononuclear leukocytes. Decreases in IP‐10 and IL‐10 transcripts were found in PEC RNA from a patient with ovarian cancer who experienced a complete response. An increase in PEC IFN‐γ transcript was seen in this same patient as well as a decrease in bFGF and VEGF expression for several weeks posttreatment. Another responding patient had increased serum and peritoneal fluid levels of IFN‐γ and TNF‐α. Of note, no cytokine production was detected in patients treated in cohorts receiving < 30 ng/kg rhIL‐12 and the majority of these patients had progressive disease. Therefore, the increase in IFN‐γ and TNF‐α levels and the decrease in IL‐10, bFGF, and VEGF transcript correlated with beneficial responses to IL‐12 therapy, as seen in other studies. While this study also suggested a decrease in IP‐10 as a potential correlate, this contradicts several other published studies that found increases in IP‐10 being indicative of tumor regression.

## 5 CORRELATES OF RESPONSE FOLLOWING IL‐12 PLASMID/GENE THERAPY

Localized administration of IL‐12 DNA has also been achieved via IL‐12 plasmid delivery by electroporation. Several groups have recently utilized this method as reported by Algazi et al.^34^ In a 2020 phase II trial by this group, the efficacy of tavokinogene teleplasmid (pIL12) was assessed in patients with stage III or IV melanoma. This study contained three patient cohorts that received i.t. plasmid injection and subsequent in vivo electroporation according to varying schedules. Cohort A (considered the main study with 28 patients) employed 0.5 mg/ml pIL12 at ¼ tumor volume followed by six pulses of in vivo electroporation at 1 second intervals with a field strength of 1300 V/cm and a pulse width of 100 µs on days 1, 5, and 8 of each 90‐day cycle. Alternative cohorts B and C, with a combined 21 patients, were given this same treatment on days 1, 8, and 15 or days 1, 5, and 8 of every 6‐week treatment cycle, respectively. Patients in cohort A seemed to respond slightly better with an overall response rate of 36% compared with an overall response rate of 30% in all cohorts. Additionally, cohort A had 5 complete responders, 5 PRs, and 12 patients with stable disease compared with cohorts B and C, which had no complete responses but 4 partial responses and 10 patients with stable disease. These cohorts were further evaluated by immune infiltrate assessment and IHC for programmed death‐ligand (PD‐L1) expression. NanoString analysis of tumor biopsy RNA was also conducted. PD‐L1 IHC staining was significantly increased in patients following treatment with pIL12 and 6 out of 8 patients who had progressive disease with pIL12 went on to respond to anti‐PD‐L1 treatment with pembrolizumab. Some responding patients also had increased total tumor infiltrating lymphocytes and an increased ratio of CD8^+^:FoxP3^+^ T regulatory cells (Tregs) in the immune infiltrates. IFN‐γ gene expression was increased only in patients experiencing clinical benefit and circulating levels of both IL‐1β and MIP‐1α were increased only in responding patients. Furthermore, systemic levels of proliferating effector memory CD8^+^ T cells and circulating cytolytic NK cells were found to be increased in responders but not in nonresponders. These correlates might be indicative of an IL‐12 induced systemic antitumor immune response. Notably, nearly half the patients in this study exhibited regression of at least one uninjected lesion.

Greaney et al.^35^ went on to further analyze patients from cohort A of this study by Algazi et al. Utilizing ELISpot, IHC, flow cytometry, and TCRβ sequencing, this team sought to elucidate any immunologic changes induced by pIL12 treatment. They found that frequencies of PD‐1^+^ and PD1^+^Ki67^+^ CD4^+^ T effector cells were significantly decreased after treatment as well as CD8^+^, PD‐1^+^CD8^+^, and PD1^+^Ki67^+^ CD8^+^ T cells. By then evaluating PBMC samples isolated from 25 patients on study for antigen‐specific responses, T cell responses to shared melanoma antigens were compared between responding and nonresponding patients. IFN‐γ responses to the antigen MAGE‐A3 were found to be significantly lower in responding patients 90 days posttreatment but no change was detected in IFN‐γ responses to antigens gp100, NY‐ESO‐1, or Melan‐A/MART‐1 between response groups. IHC also showed that CD3^+^ tumor‐infiltrating T cell percentages were higher in responding patients than nonresponding patients at day 39 posttreatment and a similar trend was found in CD3^+^CD8^+^ tumor‐infiltrating T cells, but this was not statistically significant. Moreover, posttreatment T cell infiltration correlated with circulating antigen‐specific T cells. Thus, increased intratumoral T cell frequencies posttreatment may also be considered as a marker of positive response to IL‐12 therapy.

The efficacy of i.t. delivery of IL‐12 plasmid in combination with PD‐1 blockade was assessed in a follow‐up 2020 trial that treated 23 patients with metastatic or unresectable melanoma.^36^ For this study, patient tumors were injected with tavokinogene teleplasmid (pIL12) at the same concentration and volume as the preceding study followed by 6 pulses of in vivo electroporation at 300‐ms intervals with a field strength of 1500 V/cm and a pulse width of 100 µs. This treatment was given on days 1, 5, and 8 every 6 weeks in combination with 200 mg i.v. pembrolizumab every 3 weeks. Clinical benefit was evident as 9 patients had a complete response, 2 had a partial response, and 3 had stable disease. This level of efficacy is noteworthy as 42% of patients in the study had previously experienced disease progression on PD‐1 blockade, including 1 patient with a complete response and one with stable disease. Tumor biopsies and RNA isolates were analyzed via NanoString nCounter and intratumoral IHC staining to measure gene expression/tumor immune cell infiltration related to innate and adaptive immunity. Gene transcripts associated with APCs, NK cells, proinflammatory cytokine and chemokines and T cell activation were all found to be increased post‐pIL12 treatment. However, only responding patients were found to have a treatment‐related increase in PD‐L1 expression with a concomitant increase in intratumoral mRNA IRF‐1 levels following treatment. The ratio of tumor infiltrating CD8^+^ T cells to Tregs and M2 macrophages was also elevated only in those patients who experienced a complete or partial response. Furthermore, these responding patients had significantly higher intratumoral expression of PD‐L1 posttreatment than nonresponders. Conversely, nonresponding patients had higher levels of FoxP3^+^ Tregs and reduced numbers of tumor infiltrating CD8^+^ T cells. FoxP3^+^ Tregs in nonresponding patients were also determined to be in closer proximity to CD8^+^ T cells than Tregs in responding patients by quantitative spatial analysis of multispectral IHC staining. These findings provide evidence that immune infiltrate composition and configuration can be used as a correlate for the clinical response to IL‐12 therapy.

In 2020, Bhatia et al.^37^ also explored the use of i.t. delivery of an IL‐12 plasmid (tavokinogene teleplasmid) via in vivo electroporation in patients with Merkel cell carcinoma (MCC). In this pilot study, 15 patients were divided into cohort A if they had locoregional MCC (*n* = 3), or cohort B if they had distant metastatic disease (*n* = 12). Patients in both cohorts received injections of plasmid IL‐12 at a concentration of 0.5 mg/ml followed immediately by in vivo electroporation consisting of six individual pulses of 1300 V/cm field strength with a pulse width of 100 µs in 400 ms intervals. Patients in cohort A received one cycle of treatment while cohort B patients could receive up to 4 cycles of treatment in total, at least 6 weeks apart. Two of the 3 patients in cohort A were recurrence free for over 44 months, including 1 patient with a pathologically complete response. However, the third patient had disease recurrence after 9 months. In cohort B, there were 3 PRs, 1 patient with stable disease, and 8 patients with progressive disease. Biopsies from electroporated lesions were analyzed by NanoString for changes in gene and protein expression following treatment. Increased expression of both IL‐12 protein and gene transcripts were detected in electroporated lesions following therapy. Global changes in tumor infiltrating T cells were also measured by sequencing of TCR beta chains before and after IL‐12 treatment. More specifically, due to the fact that 80% of MCC cases in the United States are virus‐positive and share viral peptide antigens (MCPyV antigens), T cell responses against these cancer‐specific conserved epitopes were evaluated. MCPyV‐specific T cell responses in tumor infiltrating leukocytes from 5 patients were measured by MCPyV‐specific tetramer staining in treated lesions, untreated lesions, and peripheral blood before and after IL‐12 therapy. Three clinically responding patients had significantly increased MCPyV‐specific tetramer^+^ staining in the treated lesion biopsies. The other two patients had either no detectable MCPyV‐specific T cell staining, or a decrease in staining after treatment. Additionally, one of the clinically responding patients also had an increase in MCPyV‐specific tetramer^+^ staining in PBMCs. Therefore, an increase in T cells against cancer‐specific conserved epitopes in both treated lesions and peripheral blood may correlate with a positive response to IL‐12 therapy.

Several other methods of localized IL‐12 administration have been developed. Heinzerling et al.^38^ conducted a phase I/II clinical trial in 2005 utilizing i.t. IL‐12 plasmid DNA delivery to patients with stage IV malignant melanoma. This team began treatment by predosing with intralesional injections of 50 µg of DNA for 14 days before splitting patients into 3 groups according to the total amount of DNA received. Each of the groups were given three intralesional injections per cycle on days 1, 8, and 15, resulting in 2 mg total DNA injected for group 1, 4 mg for group 2, and 10 to 20 mg for group 3. While only 9 patients were evaluated in this study, 1 patient receiving 10 mg of DNA did have a complete response and 2 patients experienced stable disease. Additionally, one of these patients with overall disease stabilization experienced a complete response in the injected lesion, 2 patients who experienced progressive disease had partial responses at the injection site and another progressive disease patient had disease stabilization in the injected lesion. In order to further assess the local and systemic immune responses, patient serum and tumor samples were tested for immune activation. Serum IL‐12 and IFN‐ γ levels increased in some patients following treatment but no direct correlations could be made with patient responses. Additional ELISAs for IL‐2, IL‐2R, IL‐6, and IL‐10 were also performed using patient serum but detected no significant changes after treatment. RT‐PCR analysis on tumor biopsies taken 24 h after the last DNA injection, however, found increased levels of IL‐12, IFN‐γ, and IP‐10 in responders over nonresponders. Conversely, nonresponders were found to have higher intratumoral mRNA levels of IL‐10 (induced or preexisting) than the responding patients. Patient serum was then used to assess antigen‐specific immune responses against the melanoma‐associated tumor antigens MAGE‐1 and MART‐1 by measuring tumor‐associated antibody concentrations. Both overall responding patients and injection site only responders had higher antibody concentrations than the nonresponding patients. Two nonresponding patients also had no detectable peritumoral infiltration of CD4^+^ or CD8^+^ T cells. These findings suggest a cellular and humoral response can be induced by IL‐12 gene therapy.

Thaker et al.^39^ also explored an alternative method of localized IL‐12 delivery in patients with recurrent or persistent platinum‐resistant epithelial ovarian, fallopian tube or primary peritoneal cancers. EGEN‐001 (GEN‐1) delivers the gene for functional IL‐12 protein via a synthetic DNA delivery system. It was used to deliver IL‐12 DNA in vivo by i.p. injection. GEN‐1 injections were given on days 1, 8, 15, and 22 of a 28‐day cycle at 24 mg/m2 (dose level 1) or 36 mg/m2 (dose level 2, 3). Chemotherapy was administered concurrently to these patients using pegylated liposomal doxorubicin by the i.v. route at 40 mg/m^2^ (dose level 1, 2) or 50 mg/m^2^ (dose level 3) every 28 days. Fourteen patients were evaluable for response. There were 3 patients with a partial response to treatment, 5 with stable disease, 3 with progressive disease, and 3 patients classified as having an indeterminant response. IL‐12, IFN‐γ, TNF‐α, and VEGF concentrations were assessed in plasma and peritoneal fluid samples by ELISA, however, only 5 patients had enough pretreatment samples for IL‐12 and IFN‐γ measurement and only 4 patients had enough samples measurable for TNF‐α and VEGF. While these sample sizes were small, increases in IFN‐γ and TNF‐α were found to be highest in the partial response group in both peritoneal fluid and plasma samples. VEGF levels were found to be highest in patients with progressive disease as posttreatment peritoneal and plasma levels increased by 1.7‐fold and 2.6‐fold, respectively, and decreased by approximately 50% in patients with partial responses. Increased levels of IFN‐γ and TNF‐α and decreased levels of VEGF in both the peritoneal fluid and plasma are consistent with other IL‐12 gene therapy correlative findings.

In 2019, Chiocca et al.^40^ explored the use of IL‐12 gene therapy in patients with recurrent high‐grade glioma. By using an IL‐12 expression switch inducible by delivery of an activator ligand, veledimex (VDX), they were able to control production of IL‐12 within the tumor microenvironment. To do this, an adenoviral Ad‐RTS‐mIL‐12 gene therapy vector was given intratumorally by free‐hand intraoperative injection as a single dose of 2 × 10^11^ vector particles in combination with oral VDX given once a day for 14 days. VDX was dose escalated in cohorts with 7 patients receiving 10 mg, 15 receiving 20 mg, 4 receiving 30 mg, and 6 patients receiving 40 mg. The 20 mg VDX cohort appeared to have the best response to treatment with a survival rate of 60% after 12 months compared with 0% for other cohorts. The median overall survival in the 20 mg cohort was 12.7 months with a mean follow‐up of 13.1 months. All patients were assessed for serum and cerebral spinal fluid levels of IL‐12 and IFN‐γ, CD8^+^ T cell infiltration, and both PD‐1 and PD‐L1 expression by IHC, H&E staining, and flow cytometry. Serum levels of IL‐12 and IFN‐γ increased proportionally to the dosage of VDX given and returned to baseline after discontinuation of VDX. Peak serum levels of IL‐12 ranged from 25 to 109 pg/ml and peak IFN‐γ levels ranged from 15 to 168 pg/ml across all four cohorts. A positive correlation was found between patient overall survival rate and percentage change in the ratio of CD8^+^ to FoxP3^+^ peripheral blood T cells on days 14–28 after treatment. Concurrent corticosteroid use in the overall patient population was also found to negatively affect overall survival. Thus, similar to the finding by the Algazi et al., the peripheral blood CD8^+^ to FoxP3^+^ T cell ratio may be considered a useful measure of response to IL‐12 therapy.

## 6 CONCLUDING REMARKS

The ability of IL‐12 to stimulate both innate and adaptive immune responses makes it a powerful cytokine for immunomodulation. It has the ability to promote T cell and NK cell cytotoxic activity and IFN‐γ production and exerts positive effects on myeloid cells and B cells. While the longstanding use of IL‐12 in the cancer field has generated both concerning and promising results, the improved ability to administer and control IL‐12 dosing over time has reconfirmed the potential efficacy of this cytokine therapy. The expanded use of IL‐12 therapy has created additional opportunities for correlative analyses that may help to explain the antitumor mechanism of IL‐12 and differential patient responses.

In an effort to improve this understanding, several clinical trials have collected various patient samples before and after IL‐12 treatment in order to analyze biologic variances between response groups. In this review, a collection of relevant trials was constructed to document these findings and gauge their potential to function as biomarkers of response to IL‐12 therapy. Seven potential biomarkers of response were identified in this review after being detected in at least three separate clinical trials and these are summarized in Table 1. The first biomarker identified was increased levels of IFN‐γ in plasma, serum, peritoneal fluid, or RNA transcripts following treatment with IL‐12. Increased IFN‐γ levels post‐IL‐12 were seen in 11 separate clinical trials and correlated strongly with improved patient response. This is a logical correlation as IL‐12 is a potent inducer of IFN‐γ production by activated T cells and NK cells.^41^ Elevated IFN‐γ levels may reflect an immune response to IL‐12 through activation of cytotoxic T cells and NK cells. The second biomarker identified was an increased intratumoral ratio of CD4^+^ and CD8^+^ T cells to FoxP3^+^ Tregs and M2 macrophages. This increased ratio was observed to correlate with better patient responses in seven of the clinical trials discussed. An increased activation of T cells within the tumor microenvironment and a similarly decreased population of immuno‐suppressive cells, such as Tregs or M2 macrophages are consistent with T cell‐based antitumor immunity. Without anti‐inflammatory and inhibitory signals from these suppressive populations, helper and cytotoxic T cells could exhibit increased activation allowing for improved cytotoxicity toward cancer cells.

**TABLE 1:** Summarized biomarkers of beneficial response to IL‐12 therapy including their respective locations and methods of detection

Potential correlate	Location/method of detection	Reference identified in
Increased IFN‐γ	Plasma, serum and peritoneal fluid by ELISA RNA, mRNA expression by RT‐PCR, intracellular flow cytometry Genomic DNA by cytokine genotyping assay	Gollob (16), Gollob (17), Parihar (19), Bekaii‐Saab (21), Alatrash (22), McMichael (23), Lenzi (25), Algazi (26),Heinzerling (30), Thaker (31)
Increased ratio of CD4+ and CD8+ T Cells to FoxP3+ T‐regulatory cells and M2 macrophages	Intratumoral infiltrate by flow cytometry, IHC staining	Gollob (17), Rook (24), Greaney (27), Algazi (26), Algazi (28), Heinzerling (30), Chiocca (32)
Increased IP‐10	Plasma, serum by ELISA mRNA expression by RT‐PCR	Parihar (19), Bekaii‐Saab (21), Alatrash (22), McMichael (23), Heinzerling (30)
Increased TNF‐α	Plasma, serum, peritoneal fluid by ELISA	Parihar (19), McMichael (23), Lenzi (25), Thaker (31)
Increased MIP‐1α	Plasma, serum by ELISA	Parihar (19), Bekaii‐Saab (21), Algazi (26), Algazi (28)
Increased MIG	Plasma, serum by ELISA mRNA expression by RT‐PCR	Parihar (19), Bekaii‐Saab (21), Alatrash (28)
Decreased VEGF, bFGF levels	Plasma, serum and peritoneal fluid by ELISA PEC expression by IHC staining	Younes (20), Lenzi (25), Thaker (31)

The third potential biomarker discovered was an increased expression of IP‐10 in patients following IL‐12 treatment. Five clinical trials reported this same correlate exclusively in clinically benefiting patients. IP‐10, or IFN‐γ‐inducible protein 10, is a potent chemoattractant for both NK cells and activated T cells. IP‐10 has been found to synergize with the fourth potential biomarker, TNF‐α, in a murine model of glioblastoma.^42^ Increased TNF‐α was detected in patients responding to therapy in 4 separate clinical trials. IL‐12 is known to stimulate production of TNF‐α by T cells and NK cells and combinations of IL‐12 and TNF‐α have been shown to induce CD8^+^ T cell and NK cell‐dependent antitumor immune responses in murine models of melanoma and sarcoma.^2^, ^43^ Thus, elevated TNF‐α may be reflective of a positive response to therapy due to its potential to synergize with other cytokines.

The fifth and sixth biomarkers identified were similarly increased levels of the chemokines MIP‐1α and MIG following IL‐12 therapy. Circulating levels of MIP‐1α were increased in clinically benefiting patients in 4 clinical trials, while increased MIG was detected in clinically benefiting patient plasma and serum in 3 clinical trials. MIP‐1α is a chemokine that can be secreted by a variety of cell types at sites of inflammation. MIP‐1α induces chemotaxis of leukocytes, especially T lymphocytes, which are crucial for the inflammatory response.^44^ While there is literature suggesting MIP‐1α may also play a role in tumor cell migration, its proimmune effects may be dominant in the context of IL‐12 administration. In support of this idea, Jeong et al.^45^ found that combining i.v. MIP‐1α therapy with preestablished methods of treatment for hepatocellular carcinoma, such as irradiation or sorafenib, significantly enhanced antitumor immunity dependent on increased CD8^+^, CD107A^+^, and CD11C^+^ cells. MIG is a chemokine that like IP‐10 is known to be induced by IFN‐γ. MIG plays a critical role in chemotaxis, uniquely targeting activated T cells.^46^ In previous studies by Park et al. and Tannenbaum et al.,^47^, ^48^ MIG, along with IP‐10, was found to be crucial for IL‐12‐mediated antitumor immunity. This supports the findings that MIP‐1α, MIG and the previously mentioned IP‐10 are all chemokines that may function as biomarkers of the multicell response to IL‐12 therapy.

The last potential biomarker identified in this review was the decreased RNA transcript and serum levels of the angiogenesis factors VEGF and bFGF. This effect was consistently identified in responding patients in 3 different clinical trials. VEGF is known to be an inducer of tumor angiogenesis as is bFGF.^49^ It has also been shown that increasing angiogenesis directly influences tumor volumetric growth rate, which can be largely attributed to increased secretion of VEGF and bFGF.^50^ Thus, it is understandable how decreased levels of these factors may lead to reduced angiogenesis and thus better tumor control.^51^

Future studies may also benefit from measuring the induction of negative feedback loops resulting from exogenous IL‐12 treatment. For example, the p40 subunit of IL‐12 can be produced as either a monomer or homodimer and has been shown to antagonize the effects of IL‐12 in both mice and humans.^8^, ^52^, ^53^ However, p40 has also been shown to promote macrophage chemoattraction, migration of stimulated dendritic cells, and production of IFN‐y by CD8^+^ T cells in mice.^8^, ^54^ Thus, p40 induction can exert pleiotropic effects and may be of significant interest to evaluate in response to IL‐12 therapy. In this manner, it may be possible to further delineate the antitumor mechanism of IL‐12 cytokine therapy and devise ways to make it more effective in a larger proportion of cancer patients.

While this review has highlighted several potential and plausible biomarkers of response to IL‐12 therapy, it cannot be said that these findings are conclusive. Additional studies will need to be carried out in order to substantiate these correlates as true biomarkers of response. Confirmational studies should be conducted in future trials of IL‐12 therapy, and exploratory analyses using advanced sequencing and histochemical approaches should be applied to circulating and intratumoral immune cells. The broad reliance on plasma cytokine levels in many of the studies is noted and understandable. It is expected that future studies will include a broader panel of biomarkers such as intratumoral immune infiltrate as measured by multiparameter, immune monitoring tools.

## AUTHORSHIP

*Conceptualization*: E. S. and W. E. C. *Investigation*: E. S. *Data curation‐formal analysis*: E. S. and W. E. C. *Writing—original draft*: E. S. and W. E. C. *Writing—review, editing and revision*: E. S. and W. E. C.

## DISCLOSURE

The authors state no conflict of interest.
